# Preparation, characterization, and in-vitro cytotoxicity of nanoliposomes loaded with anti-tubercular drugs and TGF-β1 siRNA for improving spinal tuberculosis therapy

**DOI:** 10.1186/s12879-022-07791-8

**Published:** 2022-11-08

**Authors:** Zongqiang Yang, Caili Lou, Xuewei Wang, Chaoran Wang, Zhiyun Shi, Ningkui Niu

**Affiliations:** 1 Department of Orthopedic, General Hospital of Ningxia Medical University, Yinchuan, 750004 Ningxia Hui Autonomous Region China; 2 Ningxia Medical University, Yinchuan, 750004 Ningxia Hui Autonomous Region China; 3 Department of Medical Experimental Center, General Hospital of Ningxia Medical University, Yinchuan, 750004 Ningxia Hui Autonomous Region China

**Keywords:** Anti-tubercular drugs, TGF-β1 siRNA, Nanoliposome, Drug delivery, Spinal tuberculosis

## Abstract

**Background:**

Tuberculosis (TB) represents a bacterial infection affecting many individuals each year and potentially leading to death. Overexpression of transforming growth factor (TGF)-β1 has a primary immunomodulatory function in human tuberculosis. This work aimed to develop nanoliposomes to facilitate the delivery of anti-tubercular products to THP-1-derived human macrophages as *Mycobacterium* host cells and to evaluate drug efficiencies as well as the effects of a TGF-β1-specific short interfering RNA (siRNA) delivery system employing nanoliposomes.

**Methods:**

In the current study, siTGF-β1 nanoliposomes loaded with the anti-TB drugs HRZ (isoniazid, rifampicin, and pyrazinamide) were prepared and characterized in vitro, determining the size, zeta potential, morphology, drug encapsulation efficiency (EE), cytotoxicity, and gene silencing efficiency of TGF-β1 siRNA.

**Results:**

HRZ/siTGF-β1 nanoliposomes appeared as smooth spheres showing the size and positive zeta potential of 168.135 ± 0.5444 nm and + 4.03 ± 1.32 mV, respectively. Drug EEs were 90%, 88%, and 37% for INH, RIF, and PZA, respectively. Meanwhile, the nanoliposomes were weakly cytotoxic towards human macrophages as assessed by the MTT assay. Nanoliposomal siTGF-β1 could significantly downregulate TGF-β1 in THP-1-derived human macrophages in vitro.

**Conclusion:**

These findings suggested that HRZ-loaded nanoliposomes with siTGF-β1 have the potential for improving spinal tuberculosis chemotherapy via nano-encapsulation of anti-TB drugs.

## Introduction

Tuberculosis (TB), due to infection by the bacterial pathogen *Mycobacterium tuberculosis* (*Mtb*), represents one of the ten most potent killers and the deadliest disease due to a single pathogen, more than HIV/AIDS, totaling 5.8 million newly diagnosed cases and approximating 1.3 million deaths in 2020 [1]. Isoniazid (INH) is employed to treat TB in combination with other anti-tubercular drugs such as rifampicin (RIF) and pyrazinamide (PZA) [2]. TB treatment is intricate because non-compliant patients complain of adverse effects of current medications, regular doses, and prolonged treatment duration [3]. Meanwhile, anti-TB drugs with low quality and limited bioavailability promote the occurrence of drug-resistant (DR), multidrug-resistant (MDR), and extensively drug-resistant (XDR) TB [4].

Spinal tuberculosis (STB) comprises 50% of all bone and joint TB cases and is the commonest extrapulmonary TB, frequently and irreversibly causing neurological damage, which results in severe socioeconomic problems [5]. STB was treated with first-line anti-TB therapeutics such as INH (H), RIF (R), and PZA (Z). Furthermore, the histopathology of TB, the pharmacokinetics of anti-TB drugs, and the drug resistance mechanism of *Mtb* have been studied in depth [6–16]. A significant difference was observed in the distribution of anti-TB drugs in STB. These drugs were at extremely low or undetectable levels in the vertebral sclerosis area and enclosed TB lesions. The conventional dosage forms of medications hardly persist in the lesion area for an extended period, making it difficult to maintain the effective drug concentration, the leading cause of prolonged recurrence observed in STB.

In the medical field, nanotechnology has led to significant improvements in cancer therapy [17], diagnostic imaging of diseases [18], tissue engineering [19], and most importantly, drug and gene delivery systems [20]. Although developing new TB molecules remains critical in curbing the TB epidemic, altering novel therapeutics in nanoparticle-based delivery systems represents a feasible, cost-effective, and readily available option [21]. Hitherto, multiple nano delivery systems for administering anti-TB products to the lung have been widely assessed and suggested alternatives to conventional TB therapy. Nanoparticles can selectively deliver into macrophages, which primarily host TB, significantly increasing the therapeutic index by enabling high drug levels where *Mtb* replicates while reducing systemic toxicity. An additional advantage is that nanoparticles for TB drugs shield them from liver catabolism and renal clearance; consequently, these products are safer and more effective than free medications, decreasing treatment time and drug resistance occurrence [2, 4, 22–24]. Therefore, developing new dosage forms of high-efficiency anti-TB drugs, improving biodistribution in diseased vertebrae, and effectively killing *Mtb* in the target tissue are critical measures for applying current anti-TB therapeutics in the treatment of STB.

In humans, transforming growth factor (TGF)-β1 plays an essential immunomodulatory role in TB [25]. TGF-β1 with excessively high activity is found in lung lavages and macrophages in individuals suffering from pulmonary TB [26, 27]. In addition, TGF-β1 potently deactivates macrophages, reducing their effectiveness in containing *Mtb* [28]. Furthermore, TGF-β1 and other cytokines (e.g., TNF-α) may be involved in tissue damage described in TB patients [29, 30]. Thus, silencing the *TGF-β1* gene by the RNA interference (RNAi) technology [31], reducing the secretion of the TGF-β1 protein in macrophages, and combining first-line anti-TB drugs to facilitate *Mtb* clearance are tools that could increase the efficacy of anti-TB medications.

Here, an anti-TB nano delivery system was engineered employing nanoliposomes as the carrier for biocompatibility and biodegradability, impressive drug loading rate, organ targeting potential, slow-release, high oral bioavailability, and prolonged half-life in circulation [32, 33]. Then, H, R, and Z were selected as first-line oral drugs for the treatment of TB. The positively-charged nanoliposomes loaded with HRZ (isoniazid/rifampicin/pyrazinamide) for the treatment of TB were successfully developed by reverse-phase evaporation and further bound to the negatively-charged siTGF-β1 to reduce the TB granuloma wrapped in *Mtb* and increase the efficacy of the drugs. Finally, the particle size, zeta potential, particle shape, and encapsulation efficiency (EE) of nanoliposomes loaded with HRZ/siTGF-β1 were characterized, evaluating their in vitro cytotoxicity as a potential alternative for the treatment of STB.

## Materials and methods

### Preparation of HRZ-loaded nanoliposomes

2,3-dioleoyl-3-trimethylammonium-Propane(DOTAP) and 1, 2-distearoyl-sn-glycero-3-hosphoethanolamine-n-[methoxy (polyethylene glycol) 2000] (DSPE-PEG 2000) were provided by Sigma-Aldrich (USA). Cholesterol, INH, RIF, and PZA (> 98% purity) were manufactured by Tokyo Chemical Industry (Japan). RPMI 1640 medium, trypsin, and fetal bovine serum (FBS) were provided by Hyclone (USA). Anti-TGF-1 (Cat No. ab 92486) was from Abcam (UK). SYBR^®^ Premix Ex Taq, PrimeScript™ RT reagent Kit with gDNA Eraser, and RNAiso Plus were manufactured by TaKaRa Biotechnology (Japan). 3-(4,5-dimethylthiazol-2-yl)-3,5-diphenyltetrazolium bromide salt (MTT) was provided by Biosharp (China). Annexin V-FITC/PI Apoptosis Detection kit and propidium iodide (PI) staining solution were provided by BD Biosciences (USA).

HRZ-loaded nanoliposomes were prepared by the thin film hydration method. Briefly, DOTAP (36 mg), DSPE-PEG2000 (50 mg), cholesterol (1 mg), INH (7.2 mg), RFP (10.9 mg), and PZA (1.8 mg) at the molar ratio 20:10:1:21:5.3:5.9 were solubilized in chloroform/methanol (4:1, v/v). After solvent evaporation (rotary evaporator, 37 °C), further drying was performed under vacuum for 1 h. The resulting inclusion complex was dissolved in 5 ml of deionized water, and a clear orange-red solution was obtained post-filtration.

The resulting nanoliposome solution was transferred into a 10 kDa ultrafiltration tube and subjected to ultrafiltration at 5000×*g* for 10 min and repeated 5 times until a colorless filtrate was obtained. The upper layer of the preserved orange-red liquid encompassed cationic liposomes containing the anti-TB drugs. Then, 10% mannitol was added to the liquid and lyophilized to obtain 67 mg of an orange-red oily HRZ-loaded nanoliposome product.

### Conjugation of HRZ-loaded nanoliposomes with siTGF-β1

Biomics Biotechnologies manufactured the siRNA oligonucleotides targeting TGF-β1 (siTGF-β1), and their sequences were as follows: siTGF-β1: sense 5′-GGA GUC AGA UCC UCA GCA AGC-3′ and antisense 5′-UUG CUG AGG AUC UGA CUC CUG-3′; non-coding control siRNA (siNC), sense 5′-GAA GGC CCA TAG CCA GTG ACT-3′ and antisense 5′-AGU CAC UGG CUA UGG GCC UUC-3′. Cationic HRZ nanoliposomes were mixed with siTGF-β1 in weight ratios of 2:1, 5:1, 10:1, and 20:1, respectively, and further underwent incubation at ambient for 30 min. The binding efficiency of the HRZ nanoliposomes with siTGF-β1 was determined by the gel retardation assay using 1.5% agarose gel (Ultrapure™ agarose, Life Technologies).

### Characterization of HRZ/siTGF-β1 nanoliposomes

The size and zeta potential of HRZ/siTGF-β1 nanoliposomes were assessed by dynamic light scattering (DLS) on a Malvern ZetasizerNano ZS (Malvern Instruments, UK) in triplicate at ambient, after dilution with double-distilled water.

The surface morphology of HRZ/siTGF-β1 nanoliposomes was assessed by transmission electron microscope (TEM) (TEM Jeol JEM-1400; JEOL, Japan). A 5:1 mass ratio of cationic nanoliposomes and siRNA was spread over a copper grid and air-dried for 30 min before detection to prepare TEM samples.

As reported previously, INH, RIF, and PZA loading in HRZ/siTGF-β1 nanoliposomes were assessed with slight modifications [34]. Briefly, the mobile phase was formulated to an optimal concentration to detect the EE of HRZ/siTGF-β1 nanoliposomes on a high-performance liquid chromatography (HPLC) system (Agilent Technologies, USA). After the loading procedure, the suspensions were submitted to centrifugation at 16,873 g for 20 min (Centrifuge 5418; Eppendorf AG, Germany). Unencapsulated drugs that remained in the supernatant were quantitated by UV detection at 334.00 nm [35].

Entrapment efficiency (%) was derived as [(weight of drug-loaded initially − the weight of unencapsulated drug)/weight of drug-loaded initially] × 100%

### In-vitro cytotoxicity assays

Human monocytes THP-1 cells provided by American Type Culture Collection (ATCC) underwent culture at 2 × 105 cells/ml in RPMI 1640 medium containing 10% FBS, penicillin (100 U/ml), and streptomycin (100 g/ml). The media were replaced twice or thrice weekly, and the cells were sub-cultured until 80–90% confluency. THP-1 cell differentiation into adherent macrophages was performed with 100 nM phorbol 12-myristate 13-acetate (PMA) for 48 h in RPMI 1640 containing 10% FBS [36]. Then, the PMA media were removed, followed by three PBS rinses, and incubated in a fresh medium for three hours. To evaluate the cytotoxic effect of the developed nanoliposomes on human macrophages, 3-(4,5-dimethylthiazol-2-yl)-2,5-diphenyltetrazolium bromide (MTT) assay was performed directed by the manufacturer. In brief, 5 × 10^3^ THP-1 cells were added to each well of a 96-well plate and allowed to differentiate into macrophages by PMA induction at 100 ng/ml for 48 h. Then, they were incubated with HRZ/siTGF-β1 nanoliposomes at 0, 5, 10, 15, 20, 25, 30, 35, 40, 45, and 50 mg/ml at 37 °C in 5% CO_2_ for 24 h. Subsequently, the medium was replaced by MTT containing culture medium. Incubation was carried out for an additional 4 h, and the reaction was stopped with an equivalent volume of DMSO for formazan crystal solubilization. Optical density was obtained at 570 nm. As described in a previous report, cell viability was quantitated, determining the percentages of viable cells and inhibitory potency (IC50) values [37]. Triplicate assays were carried out.

### Assessment of cell cycle distribution and apoptosis

Flow cytometry (FCM) was conducted to assess cell cycle distribution and apoptosis upon treatment with nanoliposomes. PMA-induced macrophages were added at 5 × 10^3^ cells per well of a 96-well plate. Upon overnight incubation, the siNC group was treated with HRZ/siNC nanoliposomes, while the siTGF-β1 groups were administered various amounts of HRZ/siTGF-β1 nanoliposomes (35 and 40 mg/ml); the HRZ group was treated with HRZ nanoliposomes. On the other hand, control cells were administered an identical volume of cell culture medium. Upon treatment, the cells underwent trypsinization, centrifugation (1000 rpm, 5 min), and staining with Annexin V-FITC/PI double-labeling kit (eBioscience, USA) before analysis for cell apoptosis. Next, cell resuspension was performed in PBS with 40 µg/ml PI followed by a 30-min incubation at 37 °C away from light to assess cell cycle distribution. After filtering through 35 µm nylon meshes, FCM on FACSCalibur (BD Biosciences) was performed for analysis. Then, the rates of apoptosis in various cell cycle phases were determined.

### Gene knockdown efficiency of TGF-β1 siRNA

THP1-derived macrophages were administered to different nanoliposomes containing HRZ, HRZ/siNC, and HRZ/siTGF-β1 (35 and 40 mg/ml) for 6 h. Total RNA from human macrophages was obtained using TRIzol, and reverse transcription was performed with PrimeScript Reverse Transcriptase Kit (TaKaRa), as directed by the manufacturer. RNA quality and amounts were assessed spectrophotometrically on a NanoDrop 1000 (Thermo Fisher Scientific). Then, qRT-PCR was carried out on an ABI PRISM Real-Time PCR system (Applied Biosystems) with the QuantiTect SYBR Green Master Mix kit (Qiagen). PCR was performed at 95 °C (10 min), followed by 40 cycles of 95 °C (5 s) and 60 °C (1 min), with the melting curve obtained at 95°. Fluorescence was collected at 60 °C every 0.3 °C until 95 °C. The primers employed were: TGF-β1, Forward 5′-GTC CTG GTG GAA TGG GTT ATA C-3′ and reverse 5′-GTT GAG TGT TCT TTG GCT TGA C-3′; GAPDH, Forward 5′‑GGT GTG AAC CAT GAG AAG TAT GA-3′ and reverse 5′-GAG TCC TTC CAC GAT ACC AAA G-3′. The 2^−ΔΔCq^ method [38] was employed to analyze triplicate assays, normalizing the data to GAPDH expression.

TGF-β1 protein amounts were determined by Western blot assays. After the treatment of THP1-derived macrophages with nanoliposomes containing HRZ, HRZ/siNC, and HRZ/siTGF-β1 (35 and 40 mg/ml), respectively, total protein was obtained with the Total Protein Extraction Kit (Bestbio, China) and quantitated by the Bradford assay (Bio-Rad, USA) as described by the manufacturer. Equal amounts of total protein were resolved by 10% SDS-PAGE. First of all, the extracted protein is added to the electrophoresis tank for electrophoresis and mold rotation, and after the transfer is completed, the band is cut and blocked according to the molecular weight size of the target protein and the marker, Rabbit polyclonal anti-TGF-β1 (Abcam) and anti-GAPDH (Wuhan Boster Biological Technology, China) primary antibodies were reacted overnight at 4 °C, followed by incubation with secondary antibodies linked to horseradish peroxidase (HRP) (Wuhan Boster Biological Technology) at ambient for 2 h. Immunoreactive bands were detected with an enhanced chemiluminescence system (Sino-American Biotechnology, China) and quantitated with Image J version 1.441 (National Institutes of Health, USA).

### Data analysis

Data are mean ± standard deviation (SD). Descriptive statistics and one-way analysis of variance (ANOVA) were performed for analysis. Independent sample Student’s t-test was carried out for group pair compassions. P < 0.05 indicated statistical significance.

## Results

### Particle size

**Fig. 1:**
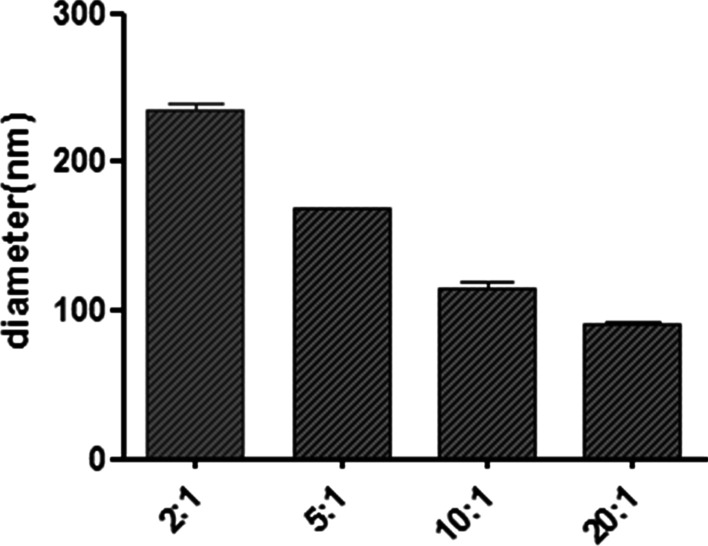
Particle size measured by DLS

DLS measured the particle size according to the principle that particles move randomly under Brownian motion. Particle size is significant in determining the cell’s absorption rate. Liposomes of about 200.00 nm could induce membrane fusion with target cells, delivering the encapsulated products into cells with high efficiency [39]. Here, the particle sizes of HRZ/siTGF-β1 nanoliposomes were determined by DLS (Table 1, Fig. 1). The product composed of HRZ nanoliposomes and siTGF-β1 with a weight ratio around 5:1 displayed an average diameter of 168.14 ± 0.54 nm, which was adequate for alveolar epithelium deposition and macrophage internalization [40]. We also found that with increasing weight ratio, the particle size of HRZ/siTGF-β1 nanoliposomes decreased from 237.89 to 91.46 nm.

**Table 1:** Mean particle size (nm) of HRZ/siTGF-β1 nanoliposomes with different weight ratios

Particle size (nm)	HRZ nanoliposomes:siTGF-β1			
	2:1	5:1	10:1	20:1
	237.885 ± 7.1912	168.135 ± 0.5444	115.265 ± 6.0033	91.46 ± 1.2445

### Zeta potential

**Fig. 2:**
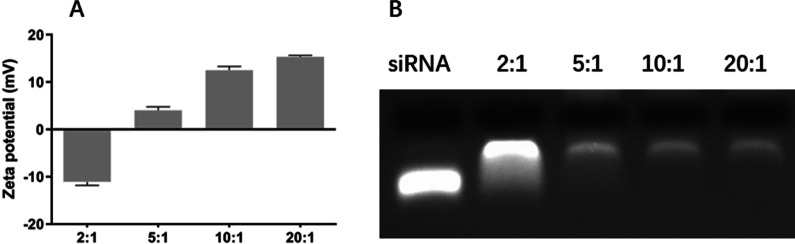
**A** Zeta potential values for different weight ratios of HRZ nanoliposomes to loaded siTGF-β1. **B** Gel retardation data for different weight ratios of HRZ nanoliposomes to loaded siTGF-β1

Zeta potential provides information regarding the electrostatic potential of the particle in solution [40]. The zeta potential of HRZ/siTGF-β1 nanoliposomes with different weight ratios was measured immediately after preparation (Table 2, Fig. 2A). The HRZ nanoliposomes carried a positive charge and absorbed the negatively-charged oligonucleotides by easily mixing the siTGF-β1 to produce the final HRZ/siTGF-β1 nanoliposomes. Zeta potential analysis revealed that the surface charge of HRZ nanoliposomes was 28.13 ± 2.40 mV in an aqueous solution. Upon conjugation of siTGF-β1 (weight ratio of HRZ nanoliposomes/siTGF-β1 = 5:1), the zeta potential was reduced to 4.03 ± 1.32 mV, thereby implying successful conjugation of the components that consumed the surface amino groups. The gel retardation assay was carried out to evaluate the siRNA loading capacity of nanoliposomes (Fig. 2B). HRZ/siTGF-β1 nanoliposomes were generated at various weight ratios of HRZ nanoliposomes to siTGF-β1 between 0:1 and 20:1. Gel electrophoresis showed a small number of bands at weight ratios > 5:1, indicating that most of the siRNA was absorbed by HRZ nanoliposomes. These findings corroborated surface charge data obtained by DLS. By adjusting HRZ nanoliposome-to-siTGF-β1, surface charges varied between 15.33 ± 0.55 mV and 28.13 ± 2.40 mV. In addition, a gradually increasing trend of zeta potential was observed with increasing amounts of HRZ nanoliposomes, in agreement with findings obtained by agarose gel electrophoresis.

**Table 2:** Zeta potential (mV) of HRZ/siTGF-β1 nanoliposomes with different weight ratios

Zeta potential (mV)	HRZ nanoliposomes	TGF-β1 siRNA	HRZ nanoliposomes:TGF-β1 siRNA			
			2:1	5:1	10:1	20:1
	28.13 ± 2.4	− 18 ± 1.77	− 11.07 ± 1.32	4.03 ± 1.32	12.5 ± 1.4	15.33 ± 0.55

Cells internalize nanoparticles with positive charges faster than neutral or negatively charged counterparts [41]. Moreover, excessive surface charge causes cell cytotoxicity [42]. This phenomenon can be prevented by ensuring an efficient loading of siRNA while maintaining a slight positive charge on the surface. Thus, a weight ratio of 5:1 for HRZ nanoliposomes to siTGF-β1 was applied in subsequent assays.

### Morphology

**Fig. 3:**
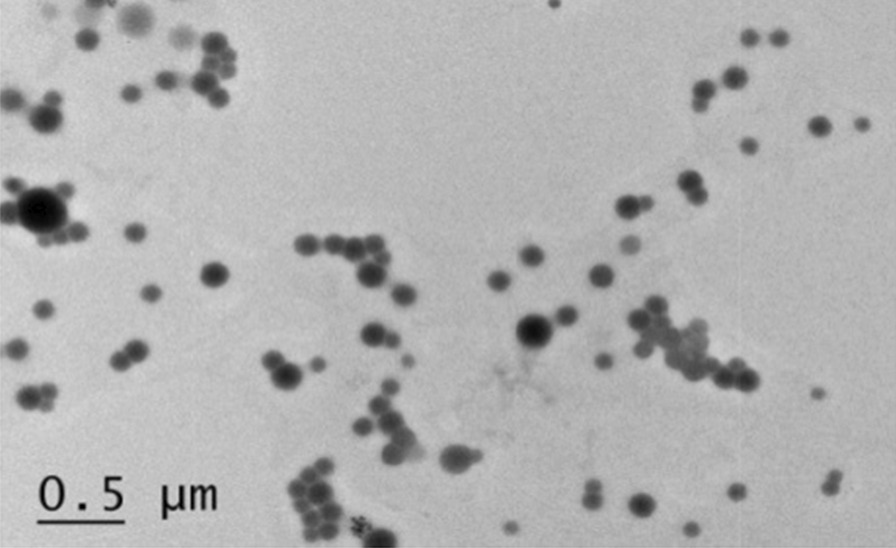
TEM images of HRZ/siTGF-β1 nanoliposomes

The appearance and morphological properties of the particles were examined by imaging air-dried HRZ/siTGF-β1 nanoliposomes under a transmission electron microscope (TEM) (Fig. 3; scale bar 0.50 µm). TEM images exhibited a spherical shape for the nanoliposomes with a homogenous surface morphology (200.00–300.00 nm), consistent with DLS data.

### EE

**Fig. 4:**
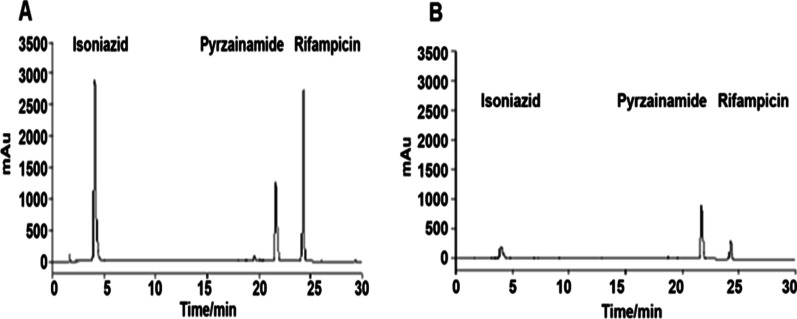
HPLC data for (**A**) pre-preparation mixture of liposomes; (**B**) filtrate after liposome preparation

EE is a valuable index concerning nano-drug delivery. Adequate drug amounts are required in a given polymer for sustained release to the target site [43]. EE (28.13 ± 2.4 mV) is calculated as the percentage amount of drug that is entrapped in the form of nanoliposomes INH, RIF, and PZA loading of HRZ nanoliposomes showed EE values of 90%, 88%, and 37%, respectively (Fig. 4A and B).

### In-vitro cytotoxicity

**Fig. 5:**
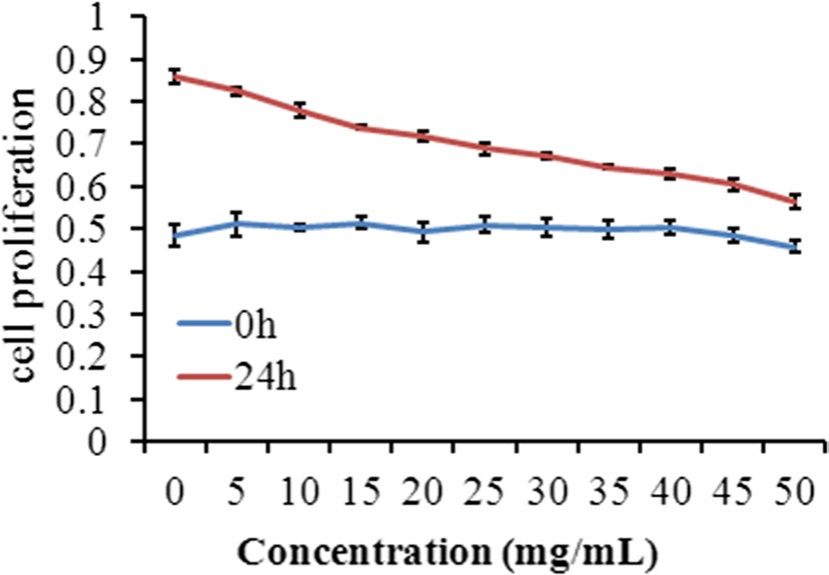
Cell viability of THP-1-derived macrophages after treatment with HRZ/siTGF-β1 nanoliposomes as assessed by the MTT assay. Notes: Survival of THP-1 derived macrophages treated with 0–50 mg/ml HRZ/siTGF-β1 nanoliposomes for 24 h. Data are mean ± SD of three independent experiments

The interaction of HRZ/siTGF-β1 nanoliposomes with macrophages was established using a human macrophage model to achieve macrophage targeting and effective concentrations of anti-TB products at the infection site. Appropriate amounts of nanoliposomes were selected for subsequent research of cytotoxicity data based on the MTT assay. This test was carried out to assess increasing concentrations (from 0 to 50.00 mg/ml) of samples compared to untreated nanoliposomes. As shown in Fig. 5, the human macrophage cell line exhibited a gradual proliferation reduction with increasing HRZ/siTGF-β1 nanoliposomes compared with untreated cells, and the IC50 value for nanoliposomes in macrophages was 37.47 mg/ml. Therefore, 35.00 and 40.00 mg/ml nanoliposomes were selected for subsequent experiments. Since nanoliposome components are considered safe or lowly cytotoxic at the levels assessed, the concentration-dependent cytotoxic effects of loaded HRZ/siTGF-β1 nanoliposomes were likely due to significant and heterogeneous particle aggregation decreasing cellular activity and promoting cell death [44]. This phenomenon could be ascribed to the toxicity imparted by the positive surface charge of nanoliposomes [45].

### Effects of nanoliposomes on cell cycle distribution and apoptosis

**Fig. 6:**
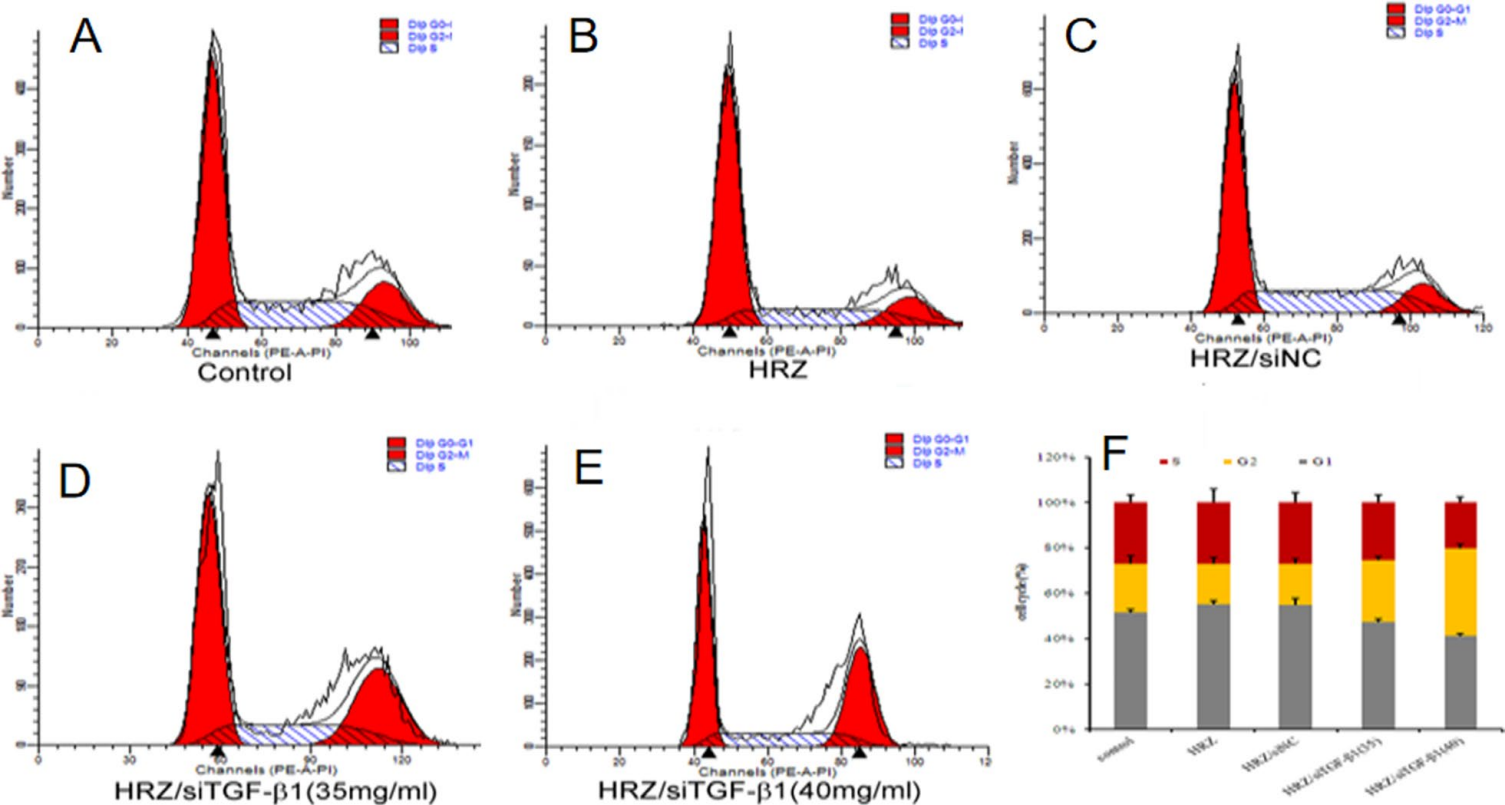
Mechanisms of HRZ/siTGF-β1 nanoliposome-mediated inhibition of macrophage growth. Cell cycle distribution in macrophages after treatment with different nanoliposomes. *P < 0.05; **P < 0.01

Macrophages were exposed to different nanoliposomes containing HRZ, HRZ/siNC, and HRZ/siTGF-β1 (35.00 and 40.00 mg/ml) for 24 h. The percentages of cells in G1, S, and G2 are shown in Table 3; live, apoptotic (early and late), and necrotic cells were also quantitated (Table 4). The results exhibited that the percentage of G2 cells significantly increased from 21.26% to 38.54% after HRZ/siTGF-β1 (40.00 mg/ml) treatment compared to 17.83% and 17.90% for cells treated with HRZ and HRZ/siNC nanoliposomes, respectively (Table 3, Fig. 6), suggesting that HRZ/siTGF-β1 nanoliposomes (40.00 mg/ml) induced cell accumulation in the G2 phase of the cell cycle in human macrophages. However, no significant differences were detected upon treatment with HRZ/siTGF-β1 nanoliposomes (35.00 mg/ml) in other cell cycle phases.

**Table 3:** Percentage of cell populations in different stages of the cell cycle following exposure to different groups of nanoliposomes for 24 h

Treatment group	G1 phase	S phase	G2 phase
Untreated cells	51.73 ± 1.17	27.00 ± 3.44	21.26 ± 3.41
HRZ	55.13 ± 1.59	27.10 ± 5.93	17.83 ± 3.01
HRZ/siNC	54.06 ± 2.93	26.90 ± 4.38	17.90 ± 2.43
HRZ/siTGF-β1 (35 mg/ml)	46.95 ± 1.15	25.20 ± 3.22	26.84 ± 1.58
HRZ/siTGF-β1 (40 mg/ml)	41.07 ± 1.22	20.37 ± 2.34	38.54 ± 2.02**

The results are presented as the average percentage of the cell population (%) ± standard deviation; P < 0.05*, *P < 0.01 *vs*. untreated cells

**Table 4:** Percentage of populations of viable and non‑viable cells exhibiting structural properties of different cell death types following exposure to different groups of nanoliposomes for 24 h

Treatment group	Viable cells	Non-viable cells		
		Necrosis	Late apoptosis	Early apoptosis
Untreated cells	91.56 ± 0.87	2.65 ± 0.16	4.52 ± 0.72	1.26 ± 0.12
HRZ	90.83 ± 1.65	1.04 ± 0.20	4.48 ± 1.31	3.64 ± 0.55
HRZ/siNC	91.36 ± 1.27	1.45 ± 0.84	3.83 ± 0.60	3.36 ± 0.32
HRZ/siTGF-β1 (35 mg/ml)	84.16 ± 1.53**	1.77 ± 0.29	9.56 ± 0.51	4.48 ± 1.28
HRZ/siTGF-β1 (40 mg/ml)	78.53 ± 1.13**	2.74 ± 0.39	12.50 ± 0.75	6.20 ± 1.16

The results are presented as the average percentage of the cell population (%) ± standard deviation; *P < 0.05 *, *P < 0.01 *vs*. untreated cells

**Fig. 7:**
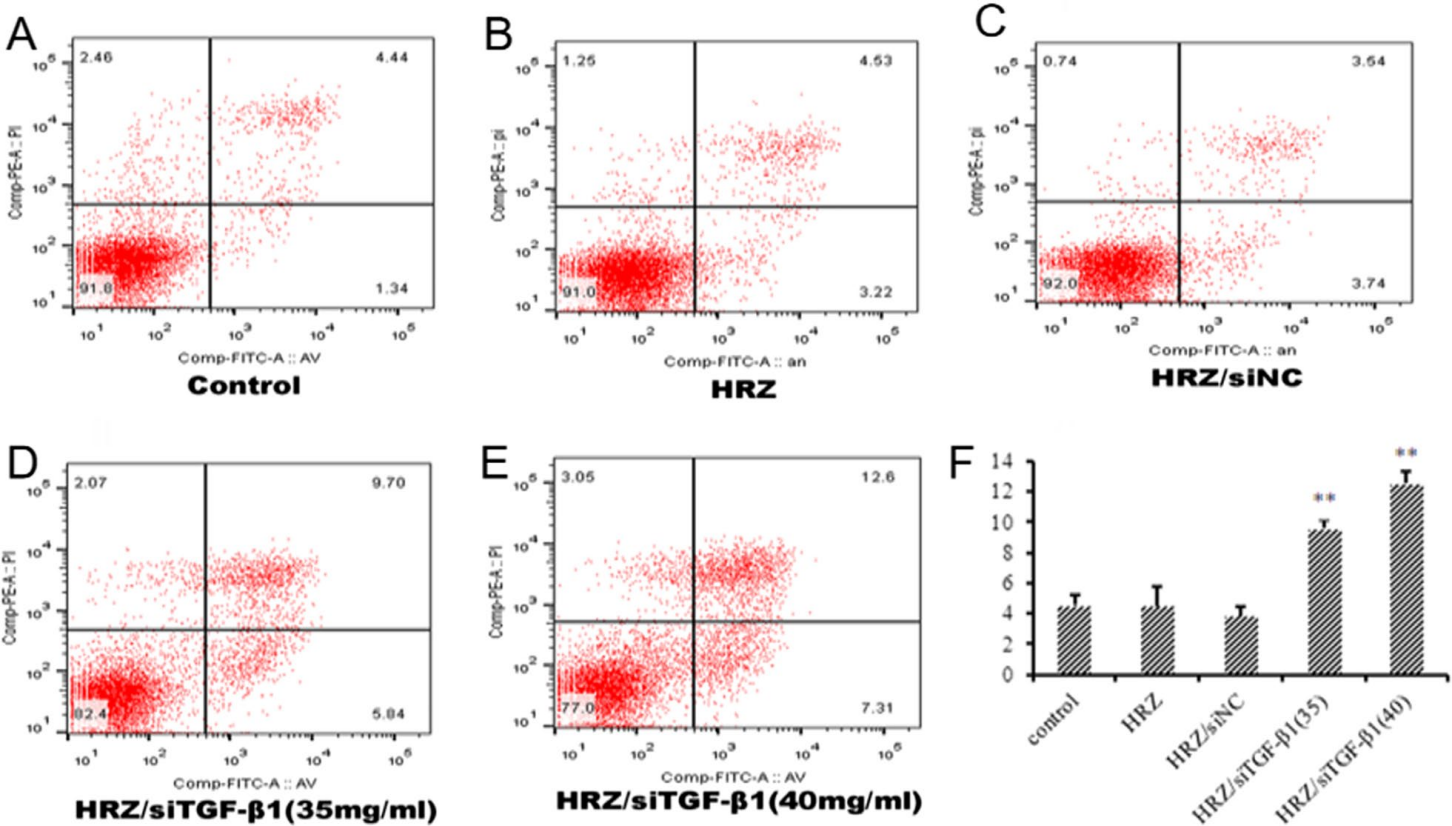
Mechanisms of HRZ/siTGF-β1 nanoliposome-mediated inhibition of macrophage growth. Apoptosis in macrophages after treatment with different nanoliposomes. *P < 0.05; **P < 0.01

Moreover, cells treated with HRZ/siTGF-β1 had a slightly higher apoptotic rate than untreated cells (Table 4, Fig. 7). At the same time, no apparent cytotoxicity was observed for HRZ and HRZ/siNC nanoliposomes, which might be due to the high uptake of siTGF-β1. These findings suggested that HRZ/siTGF-β1 nanoliposomes possessed adequate biocompatibility, although TGF-β1 siRNA conjugation slightly increased cytotoxicity.

### TGF-β1 silencing analysis

**Fig. 8:**
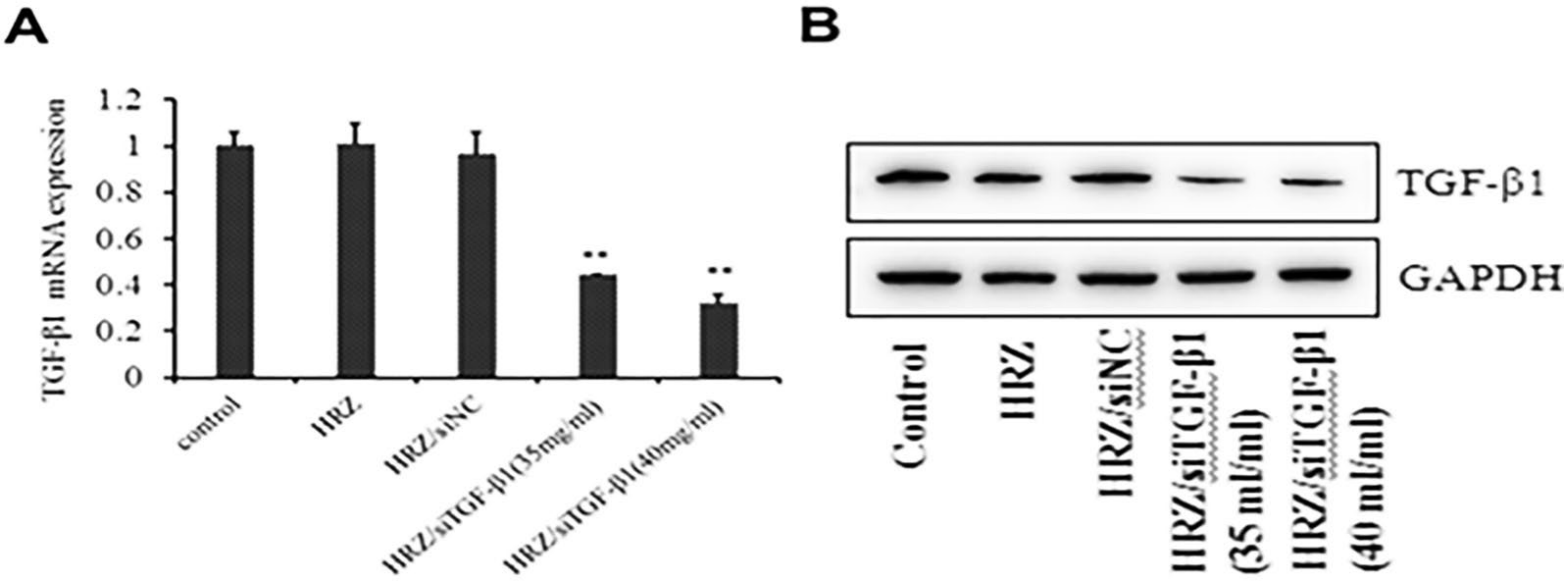
Effects of TGF-β1 knockdown measured by (**A**) qRT-PCR and (**B**) Western blot. siTGF-β1, siRNA targeting *TGF-β1* mRNA; HRZ, control group of HRZ nanoliposomes; siNC, non-coding siRNA control group; Control, PBS group. *P < 0.05; **P < 0.01

Total mRNA and protein were obtained 24 h after transfection from human macrophages treated with or without HRZ/siTGF-β1 nanoliposomes to confirm the knockdown efficiency of siTGF-β1. Negative and blank control cells were treated with non-coding siRNA (siNC) and PBS. As shown in Fig. 8, TGF-β1 mRNA and protein amounts were markedly reduced in the HRZ/siTGF-β1 group compared with the negative and blank control groups. Although the resolution of the picture is not high, it does not affect the experimental results. These data indicated that siTGF-β1 successfully repressed TGF-β1 expression at the gene and protein levels.

## Discussion

STB also termed Pott’s disease, encompasses 50% of all musculoskeletal TB cases [46]. Left untreated causes paraspinal abscesses, spinal cord compression, spine deformities, and neurological defects [46, 47]. Severe bone TB can be effectively treated by combining surgery with anti-TB drugs administered for an optimal duration [48–50]. According to WHO guidelines, long-term administration of anti-TB multiple medicines is essential for treating bone TB [51]. However, high dosages of anti-tubercular products are necessary to achieve effective concentrations at target sites due to limited permeability and metabolism [52, 53]. Innovative anti-TB drug delivery biomaterials have tremendous potential for treating STB and could achieve high drug concentrations at the target site with reduced drug amounts throughout the body, markedly reducing toxicity [54, 55]. Therefore, we developed nanoliposomes that encapsulated first-line anti-TB medicines, i.e., INH, RIF, and PZA, and conjugated them to TGF-β1 siRNA. Multiple properties verified the successful production of nanoliposomes, and the end-products were evaluated for drug encapsulation efficiency, cytotoxicity, and TGF-β1 siRNA silencing effects in THP-1-derived human macrophages.

Particle shape significantly affects cellular uptake, distribution within the cell, and cytotoxicity. Nanoparticles are taken up according to the following order based on shape: sphere > cube > rod > disk; this is likely because the cell membrane is flexible around low-aspect-ratio particles [40, 56]. Microscopy revealed a spherical shape of the engineered nanoparticles, with the particle size ranging from 100 to 200 nm, which allows a wide distribution in most organs [57]. Another critical parameter is zeta potential, which depicts the charge and stability of the prepared nanoparticles [58]. Reportedly, a high surface charge reduces the aggregation of particles [59]. The degree and rate of macrophage uptake show direct associations with particles’ net charge; the physiological compatibility of a negatively charged surface is greater than that of the positively charged counterpart [40]. Moreover, localization in lysosomes, where *Mtb* survives, is more pronounced in negatively charged particles than in positively charged ones [60]. In the present study, the zeta-potential values of HRZ nanoliposomes and TGF-β1 siRNA were 28.13 and -18 mV, respectively, whereas those of HRZ/siTGF-β1 nanoliposomes with different weight ratios ranged from − 11.07 to 15.33 mV.

EE in liposomes is impacted by various parameters, including the preparative method and the features of liposomes and loaded molecules [61]. Hydrophobic and hydrophilic substances have high (reaching 100%) and low EE values [62]. Substances with intermediate hydrophilicity and lipophilicity generally distribute between the water and lipid phases, and any solubility alteration affects their partitioning, thereby modifying the EE [61]. In this study, the 100.00–200.00 nm HRZ/siTGF-β1 nanoliposomes showed high drug encapsulation efficiencies of 90%, 88%, and 37% for INH, RIF, and PZA, respectively.

Nanoparticles have potential toxicity to the liver, kidney, neurons, and cardiovascular system, which would limit their application in the clinic. Therefore, reducing nanoparticle quantities is preferable, and low-toxicity or concentration particles should be utilized [63]. The MTT assay and FCM indicated that HRZ/siTGF-β1 nanoliposomes had low cytotoxicity and were concentration-dependent in human macrophages. Interestingly, cell cycle distribution in THP-1-derived macrophages was unaltered upon drug administration at 35 mg/ml. In comparison, the cells treated with HRZ/siTGF-β1 at a 40 mg/ml concentration showed a higher apoptotic rate than untreated cells.

Macrophages are critical cells in the immune response against *mycobacteria* and provide a niche for *Mtb* replication [64, 65]. Coordinated events among immune factors, especially macrophages and T cells, play essential roles in inhibiting TB infection [26]. In addition, macrophages and T cell functions are regulated mainly by local cytokines, necessary for developing immune reactions against *Mtb*. Several reports revealed that elevated TGF-β1 amounts suppress immune responses targeting *Mtb* by modulating proliferation, differentiation, and functions in particular immune cells [25]. In addition, TGF-β1 is expressed in non-necrotizing granulomas of sarcoidosis and TB granulomas [27, 66, 67]. Thanks to TGF-β1’s essential function in TB pathogenesis, this infection could be controlled by TGF-β1 suppression while administering anti-TB drugs. Currently, siRNA-mediated gene knockdown is considered a robust approach for reducing aberrantly elevated amounts of target genes, rendering it putative for clinical therapy [68]. The wide application of siRNAs for treatment is based on well-designed systems delivering siRNAs into target cells with high efficiency [69]. Nanoliposomes efficiently carry and deliver siRNAs in vivo [70]. In this study, TGF-β1 siRNA-mediated gene knockdown downregulated TGF-β1, decreasing the formation of tuberculous granulomas. The above data showed that the developed HRZ/siTGF-β1 nanoliposomes significantly reduced TGF-β1 mRNA and protein expression levels in THP-1-derived macrophages.

Collectively, HRZ (anti-TB drugs INH, RIF, and PZA)-loaded nanoliposomes with siTGF-β1 were successfully developed with high encapsulation efficacy and characterized by a spherical shape within nanometer size. These formulations had low cytotoxicity and potent *TGF-β1* gene silencing, laying the foundation for in vivo studies.

In conclusion, the urgency of effective treatment of TB, which is among the nine leading causes of death worldwide, was tackled by developing nanoliposomes to deliver anti-TB drugs directly to the infection site. We successfully developed nanoliposomes loaded with HRZ, followed by TGF-β1 siRNA encapsulation in the present work. These nanoliposomes were in the nanometer range, with a diameter averaging 168 nm as determined by DLS. Additionally, they had elevated zeta potential, suggesting high physical stability. Morphologically, they were spherical and uniform, with a smooth surface. INH and RIF had elevated encapsulation percentages, with > 80% drug encapsulation efficiencies. Finally, the developed nanoliposomes had low cytotoxicity and affected the viability of THP-1-derived human macrophages in a concentration-dependent manner. Overall, the novel nanoliposomes exhibited potential as excellent vehicles for delivering drugs, e.g., antituberculous medicines. This system would significantly impact the design of therapeutic regimens, improving patient compliance. Nevertheless, these nanoliposomes should be further investigated in animal models to obtain supportive in vivo data for potential clinical applications in the future.

## Data Availability

All data generated or analyzed during this study are included in this published article [and its additional information files].
